# Evaluation of Rpoβ Gene and Its Various Mutants in Multidrug-Resistant Tuberculosis Cases by GeneXpert Method

**DOI:** 10.7759/cureus.31249

**Published:** 2022-11-08

**Authors:** Jayalakshmi S, Harapriya Kar, Anahita V. Bhesania Hodiwala, Snehal Wagh, Manoj S Patil

**Affiliations:** 1 Department of Microbiology, Mahatma Gandhi Mission (MGM) Medical College and Hospital, Navi Mumbai, IND; 2 Department of Research and Development, Jawaharlal Nehru Medical College, Datta Meghe Institute of Medical Sciences, Wardha, IND

**Keywords:** multidrug-resistant tuberculosis, mdr-tb, genexpert method, mutants, rpoβ gene

## Abstract

Introduction: India is one of the countries in the world which contribute to the global burden of multidrug-resistant tuberculosis (MDR-TB). GeneXpert is a method recommended by the World Health Organization (WHO) that uses five overlapping probes (Probe A to E) to detect mutation in the beta subunit of ribonucleic acid (RNA) polymerase gene (Rpoβ) responsible for rifampicin resistance in *Mycobacterium tuberculosis *(MTB).

Method: All the pulmonary and extrapulmonary samples received in tuberculosis (TB) laboratory for testing of MTB from January 2018 to December 2020 were analyzed for bacillary load and rifampicin resistance was identified by analyzing the number of missing probes.

Results: During the study period, a total of 10,021 samples were tested for MDR-TB. Out of those samples, 2674 samples were positive for MTB of which 2321 were pulmonary samples and 353 were extra-pulmonary samples. Rifampicin resistance was detected in 385 pulmonary samples and 63 extrapulmonary samples. These samples were further differentiated according to the bacillary load. The highest number of mutations was observed in Probe E followed by Probe B, Probe A, and Probe D, and the lowest was observed in Probe C. Also, mutations were associated when all probes were present or a few combinations of probes were missing.

Conclusion: GeneXpert assay is a rapid molecular method that detected MTB and rifampicin resistance with a two-hour turnaround. It uses molecular beacons to detect mutation in the Rpoβ gene. This study can be useful in analyzing the prevalence and epidemiology of MTB in a particular demographic area and also the addition of a few more probes can enhance the identification of the mutation in other codons and therefore better therapeutic interventions can be developed accordingly.

## Introduction

Tuberculosis (TB) is one of the ancient and deadliest diseases. It is curable yet possesses a great threat to mankind. According to the World Health Organization (WHO), around nine million cases are reported every year worldwide. At least 1.5 million deaths occur worldwide every year due to TB [1-3]. India is one of those countries contributing a major part to the global burden of TB. About one-third of prevailing global cases are from India. More than two million cases are recorded every year in India, and about 400,000 people died due to TB in the year 2017 [4], but with recent therapies, more than six million people were saved from death in the past 15 years [5]. However, another challenge of multidrug-resistant TB (MDR-TB) came into the act which incorporated a great burden on the healthcare system [5].

Due to a lack of improvement in the diagnostic tools, most TB cases remain undetected and diagnosis of MDR-TB is usually hampered [5,6]. Recent advancement in diagnostic techniques, which includes Mycobacteria Growth Indicator Tube (MGIT), Nucleic Acid Amplification Test (NAAT), Line Probe Assays (LiPAs), and Polymerase Chain Reaction (PCR), has led to the detection of the majority of cases that are missed through conventional techniques along with the detection of MDR or Extensive Drug Resistant (XDR) cases [6,7]. One such rapid method is the GeneXpert recommended by WHO which detects *Mycobacterium tuberculosis* (MTB) along with rifampicin (Rif) resistance in pulmonary as well as extrapulmonary samples. It works on the principle of semi-nested Reverse Transcription - Polymerase Chain Reaction (RT- PCR) with a turn-around time of two hours. It detects Rif resistance determining region (81 bp -RRDR) hotspot mutation region of the beta subunit of ribonucleic acid (RNA) polymerase gene (ropβ) towards rifampicin. It has a sensitivity of 95% [8-10]. There are five overlapping probes (A to E) present in the system. When two or more probes impart signals, it indicates the presence of MTB. If at least one of five probes is missing then it is considered to be Rif-resistant. Also, if the difference between the first and last cycle threshold (Ct) value is more than or equal to four, the patient is considered to be Rif-resistant [11].

Rapid detection of this infectious disease along with patient awareness and proper drug regime will contribute greatly to the End TB 2025 Campaign organized by the Government of India. Therefore, this study was undertaken to acknowledge the drug resistance pattern of MTB according to missing sequential probes and the Ct values of patients attending a tertiary care hospital (TCH) in Navi Mumbai, India.

## Materials and methods

After seeking Institutional Ethics Committee approval with reference number N-EC/2022/02/13, a retrospective study was conducted on all pulmonary and extrapulmonary samples received in Tuberculosis Laboratory, Mahatma Gandhi Mission (MGM) Medical College and Hospital, Navi Mumbai, India, through Directly Observed Therapy Short Course (DOTS) Centre from January 2018 to December 2020. Samples were analyzed from patients with a history of TB, clinically suspected of TB, or any previous exposure to key populations having MDR-TB. All the samples were subjected to GeneXpert MTB/Rif assay (Cepheid, Sunnyvale, California, United States). Retrospective data were examined in databases and preserved logbooks. Clinical and socio-demographic information was examined for patients who had Rif-resistant TB (RR-TB) findings. The database was accessed to get crucial information.

Sputum samples as well as extrapulmonary specimens such as lymph node aspirates, pleural fluid, pus, abscess, ascetic fluid, and bronchoalveolar leverages (BAL) were obtained from TB suspects. Approximately 4 ml of sputum and 8 ml of sample reagent buffer (included in the kit) were mixed, and the tubes were vigorously shaken 20 times before being left for 10 minutes. Following this, it was mixed again and stayed for five minutes. Later, approximately 2 < ml < 4 (more than 2 ml but less than 4 ml) of the specimen was dispensed into Xpert MTB/RIF’s cartridge and loaded into the GeneXpert instrument. The result was released after two hours. For extrapulmonary samples, different approaches were used based on the nature of the specimen. For example, lymph node samples were decontaminated by using a 3% N-acetyl-l-cysteine-sodium hydroxide (NALC-NaOH) method, and the sediment was used for the test in 1:3 ratios (0.5 ml sediment and 1.5 ml sample reagent buffer).

Results from Xpert MTB/Rif assay were categorized into three result types: MTB not detected, MTB detected, and Error/Invalid/No results. Along with all MTB detected test results, Rif resistance was determined.

Results were analyzed for Successful and Unsuccessful (Error or No results) samples. All the data were entered in Excel Sheet and graphs/ tables were made accordingly. Bacterial load distribution was observed for all the positive samples irrespective of MDR and was termed as Very Low, Low, Medium, and High. Those samples which were positive with Rif resistance were further analyzed for the number of missing probes indicating mutation leading to MDR.

## Results

During a three-year study period, a total of 10,021 samples were tested using MTB GeneXpert assay. Out of these tested samples, MTB was detected from 2674 samples, and 6944 samples were negative. Unsuccessful results due to ‘error’ and ‘no result’ were 313 and 91 respectively. Of these 404 unsuccessful samples, 362 were subjected to repeat testing while the rest samples could not be re-tested due to the unavailability of the second sample or insufficient sample. The GeneXpert MTB data revealed that out of the total samples, MTB was not detected in 69% and was detected in 27%, whereas 3% showed invalid results and 1% showed no results.

Of all the MTB detected samples, 2321 were pulmonary specimens including Sputum, Gastric Lavage, and Bronchioalveolar Lavage (Table 1). Of these positive pulmonary samples, Rif-sensitive were 1936 (83.41%) while 385 (16.59%) were Rif-resistant.

**Table 1:** GeneXpert results of pulmonary samples MTB = *Mycobacterium Tuberculosis*

Specimen	MTB Detected	Rifampicin Resistant (Rif-Resistant)
Sputum	2285	378
Gastric Lavage	14	3
Bronchioalveolar Lavage	22	4

MTB detected in extrapulmonary specimens (Fine Needle Aspiration Cytology [FNAC], Cerebrospinal Fluid [CSF], Tissue, Pus, Lymph Node, Pleural Fluid) were 353 out of which 290 (82.15%) were Rif-sensitive and 63 (17.85%) were Rif-resistant (Table 2). 

**Table 2:** GeneXpert results of extrapulmonary samples MTB = *Mycobacterium Tuberculosis;* FNAC = Fine Needle Aspiration Cytology; CSF = Cerebrospinal Fluid

Specimen	MTB Detected	Rifampicin Resistant (Rif-Resistant)
FNAC	74	10
CSF	21	1
Tissue	31	6
Pus	74	13
Lymph Node	111	24
Pleural Fluid	42	9

Therefore, these positive samples were then further analyzed. Few samples showed indeterminate results for Rif resistance, which were then retested. Positive samples, both pulmonary and extrapulmonary, samples were differentiated according to bacterial load in the GeneXpert assay (Table 3).

**Table 3:** Bacterial load distribution in samples

Specimen	Very Low	Low	Medium	High
Sputum	290	494	750	650
Gastric Lavage	05	03	03	03
Bronchoalveolar Lavage (BAL)	04	11	06	01
Fine Needle Aspiration Cytology (FNAC)	28	35	08	03
Cerebrospinal Fluid (CSF)	13	07	01	00
Tissue	15	14	02	00
Pus	09	43	19	03
Lymph Node	53	46	10	02
Pleural Fluid	15	19	07	01

All the samples showing Rif resistance were then analyzed according to the number of probes missing. Amongst all the probes, the highest mutation was seen in Probe E (67.63%) followed by Probe B (6.02%), Probe A (4.24%), Probe D (3.79%), and the least was in Probe C (0.22%). Also, a few specimens were missing a combination of probes like DE (1.56%), AB (1.11%), BD (0.44%), and ADE (0.44%). Also, 67 (14.95%) Rif resistance samples showed no mutation in any probe and were having a Ct interval of more than 3.5 (Table 4).

**Table 4:** Mutation in probes

Specimen	All probes present	Probe A	Probe B	Probe C	Probe D	Probe E	Probes BD	Probes AB	Probes DE	Probes ADE
Sputum	56	19	23	01	12	252	02	05	07	01
Gastric Lavage	00	00	02	00	00	03	00	00	00	00
Bronchoalveolar Lavage (BAL)	00	00	00	00	00	04	00	00	00	00
Fine Needle Aspiration Cytology (FNAC)	02	00	00	00	00	08	00	00	00	00
Cerebrospinal Fluid (CSF)	01	00	00	00	00	00	00	00	00	00
Tissue	01	00	02	00	00	03	00	00	00	00
Pus	00	00	00	00	02	11	00	00	00	00
Lymph Node	03	00	00	00	02	19	00	00	00	00
Pleural Fluid	04	00	00	00	01	03	00	00	00	01

## Discussion

In this study, Xpert MTB/Rif assay was used to understand the commonness of mutation leading to resistance against Rif in MTB cases. GeneXpert assay is a WHO-recommended RT-PCR technique that has three specific primers present that amplify a part of the Rpoβ gene with a core containing 81 base pairs.

In this assay, five molecular probes are used i.e., Probe A to E. Fluorophores are used to label these molecular beacons. These probes depict mutation in codons leading to Rif resistance. The Ct value of probes varies with a maximum threshold of 39.0 and 36.0 for Probe A, B, C, and Probe D, E respectively [12]. As of now, beyond 50 mutations have been reported in RRDR of the Rpoβ gene leading to Rif resistance, which may vary to higher levels or lower levels. A higher level of Rif resistance occurs due to point mutation in Probe B (Codon 513), Probe E (Codon 531), and Probe D (Codon 526). A lower level of resistance occurs due to mutation in Probe A (Codon 511), Probe B (Codon 516 & 518), Probe C (Codon 522), and Probe E (Codon 533) [13].

In this study, from the samples altogether tested, 27% of MTB was detected in which 23.16% of pulmonary samples were Rif-sensitive while 16.75% were Rif-resistant. Also, 3.52% of extrapulmonary samples were Rif-sensitive and 0.62% were Rif-resistant. These obtained results were fairly corresponding to the data given by WHO [14]. The mutations responsible for Rif resistance were highest in Probe E followed by Probe B, Probe A, Probe D, Probes DE, Probes AB, Probes ADE, and Probe BD, and the least was Probe C. Also, Rif resistance was seen when no probes were missing.

Analysis of our study was done after comparing it with four other studies (Table 5) [15-18]. The comparison showed that in the first three studies the mutations were seen highest in Probe E followed by Probe D, Probe B, and Probe A, and least in Probe C, while in the last study, the pattern of missing probes were corresponding to the results obtained in our study. The mutation was highest in Probe E followed by Probe B, Probe D, Probe A, and Probe C. It shows that developing countries like India, Bangladesh, and Pakistan have a similar kind of mutation in MTB [18].

**Table 5:** Comparison of studies done around the world MTB = *Mycobacterium tuberculosis*

Study	Country	Total MTB Detected	Total Rifampicin Resistance Detected	Probe A %	Probe B %	Probe C %	Probe D %	Probe E %	% Of All Probes Present	% Of Combination of Probe
Current Study	India	2674	448	4.24	6.02	0.22	3.79	67.63	67.63	3.57
Reddy and Alvarez-Uria (2017) [16]	India	1851	171	8.18	15.20	0.58	18.12	54.97	2.92	0.58
Alemu et.al. (2020) [16]	Ethiopia		100	-	3	-	10	81	-	6
Mboowa et.al. (2014) [17]	Uganda	1501	12	8.33	25	-	8.33	58.33	-	-
Alamgir et.al. (2021) [18]	Pakistan		713	4.06	14.30	1.54	11.07	63.67	-	2.52

Analysis of data was done using IBM SPSS Statistics for Windows, Version 20.0 (Released 2011; IBM Corp., Armonk, New York, United States). Descriptive statistics were documented using the Chi-square test which demonstrated that Probe E is associated with both high and low resistance but Probe D is associated only with a high level of resistance. Our study shows that majority of the mutations are seen in Probe D after Probe E, which indicates bacteria with a higher level of Rif resistance. According to the current study as well as other studies, the least mutation is seen in Probe C, which may be either due to reduced selection pressure or reduced susceptibility to this region.

Apart from the missing single probe, mutations in multiple probes have also been reported. This can be associated with the ability of bacteria to develop resistance by adapting to drug exposure. Also, resistance was reported when all the probes were present. All these factors can contribute to providing information to develop and improve molecular techniques and assess the trend of MDR in bacteria [19]. The bacillary load plays a major role in determining the Rif resistance in bacteria. It helps in understanding the level of MDR, which in turn, helps in providing adequate treatment, stopping from developing further resistance, and reducing transmission. A low level of bacillary load is associated with false detection of Rif resistance or indeterminate results [20].

In our study, very low and low bacillary loads were majorly reported in extrapulmonary samples as they are considered to be paucibacillary in nature. Also, pulmonary samples were having a very low and low bacillary load, which can indicate a recent infection. Whereas medium and high bacillary loads were seen in pulmonary samples as compared to that of extrapulmonary samples. Also, in this study, the error rate of the samples was around 3-4%. A similar study showed an error rate of 3.28% [21]. Also, a similar type of the previous study gave an error rate of 4.5% [22]. The error rate is comparatively higher in extrapulmonary samples as compared to the pulmonary samples because of the invasive procedures used while collecting the samples. The blood present in these specimens acts as an inhibitor leading to the error [19-21].

Therefore, this GeneXpert MTB/RIF assay is one of the most leading molecular techniques which is endorsed by WHO in the detection of MDR-TB. This method uses a combination of probes to detect the mutation leading to Rif resistance. But there are a few limitations and drawbacks, such as it cannot detect mutation in a particular codon or low bacillary load does not allow for differentiation between mutated and wild-type sequences [20]. Hence, the addition of a few more probes and continuous study on these mutations can help the GeneXpert assay to provide more accurate results and prevent the transmission of TB.

## Conclusions

In this study, the GeneXpert assay was used to detect the Resistance to Rif in patients suffering from TB. The five molecular probes were assessed to understand the pattern of mutation in the RRDR region of the Rpoβ gene. Out of all the samples tested, 27% of the samples were positive for MTB, out of which 16.75% were Rif-resistant.

The highest mutation was observed in codon 529-533, i.e., Probe E followed by Probe B, Probe A, and Probe D, and least in Probe C. There were mutations observed in the combination of probes and also when all probes were present with Ct cycle value more than four. Also, the bacillary load was seen to be associated with the level of Rif resistance.

Therefore, this study provides insight into the epidemiology of mutation in different codons of MTB complex as the trend of missing probes varies in different regions. Also, this study can be compared with the gold standard methods to understand the pattern of resistance and hence help in preparing better therapeutic interventions and improved molecular techniques to identify and assess the MTB complex in near future.
